# *Trypanosoma cruzi* antigen detection in blood to assess treatment efficacy and cure in mice models of Chagas disease

**DOI:** 10.3389/fimmu.2023.1340755

**Published:** 2024-01-12

**Authors:** Fernanda Fortes de Araujo, Rana Nagarkatti, Ana Lia Mazzeti, Karolina Ribeiro Gonçalves, Lívia de Figueiredo Diniz, Isabela Campos do Vale, Olindo Assis Martins-Filho, Alain Debrabant, Maria Terezinha Bahia, Andréa Teixeira-Carvalho

**Affiliations:** ^1^ Grupo Integrado de Pesquisas em Biomarcadores, Instituto René Rachou, Fundação Oswaldo Cruz, Belo Horizonte, Brazil; ^2^ Laboratory of Emerging Pathogens, Division of Emerging and Transfusion Transmitted Diseases, Office of Blood Research and Review, Center for Biologics Evaluation and Research, Food and Drug Administration, Silver Spring, MD, United States; ^3^ Laboratório de Doenças Parasitárias, Escola de Medicina, Departamento de Ciências Biológicas & Núcleo de Pesquisas em Ciências Biológicas, Universidade Federal de Ouro Preto, Ouro Preto, Brazil; ^4^ Laboratório de Parasitologia Básica, Programa de Pós-Graduação em Ciências Biológicas, Instituto de Ciências Biomédicas, Universidade Federal de Alfenas, Alfenas, Brazil

**Keywords:** Chagas disease, *Trypanosoma cruzi*, aptamer, treatment, efficacy

## Abstract

**Introduction:**

Chagas disease (CD) is caused by the protozoan parasite *Trypanosoma cruzi*. Although endemic mainly in Latin America, CD has become a global public health problem due to migration of infected individuals to non-endemic regions. Despite progress made in drug development, preclinical assays for drug discovery are required to accelerate the development of new drugs with reduced side effects, which are much needed for human treatment.

**Methods:**

We used a cure model of infected mice treated with Fexinidazole (FZ) to further validate a novel Enzyme Linked Aptamer (ELA) assay that detects parasite biomarkers circulating in the blood of infected animals.

**Results:**

The ELA assay showed cure by FZ in ~71% and ~77% of mice infected with the VL-10 and Colombiana strains of *T. cruzi*, respectively. The ELA assay also revealed superior treatment efficacy of FZ compared to Benznidazole prior to immunosuppression treatment.

**Discussion:**

Our study supports the use of ELA assay as an alternative to traditional serology or blood PCR to assess the efficacy of antichagasic drugs during their preclinical phase of development. Further, the combination of high sensitivity and ease of use make this parasite antigen detection assay an attractive new tool to facilitate the development of much needed new therapies for CD.

## 1 Introduction

Chagas disease (CD) is caused by the protozoan parasite *Trypanosoma cruzi* (Chagas, 1909) (Kinetoplastea: Trypanosomatidae) (1). Although endemic mainly in Latin American countries, CD has become a global public health problem due to migration of infected individuals to non-endemic regions. It has been estimated that more than 7 million people worldwide are infected with *T. cruzi*, with approximately 25 million at risk of infection (2).

The current treatment options for CD are limited to nifurtimox (NF) (Lampit®/Bayer) and Benznidazole (BZ) (LAFEPE and Abarax®/ELEA) (3). These compounds are active in the acute phase of the disease with treatment efficacy up to 80% (4). Benznidazole is highly effective in treating the acute and early chronic phases of CD (3–5). However, the benefits of BZ treatment during the chronic phase did not reach a consensus yet (6). The recent BENEFIT clinical trial showed that BZ treatment of patients with established Chagas cardiomyopathy was able to reduce (transiently) blood parasite levels but did not reduce cardiac clinical deterioration through 5 years of follow-up (7). On the other hand, pre-clinical studies have demonstrated the beneficial effect of etiologic treatment on reducing tissue damage and on electrocardiographic alterations (8–11). Additionally, BZ treatment was able to induce a mild improvement in systolic dysfunction in dogs chronically infected with *T. cruzi* (12). Clearly, the evidences show divergent effects of BZ during the chronic phase and are associated with its severe side effects and toxicity. Thus, the development of new specific treatments for CD is essential.

The low tolerance of the NF and BZ results in the discontinuation of treatment in patients (13, 14). More importantly, the treatment regimen with these drugs does not result in sterile cure in patients. Despite some progress being made in preclinical drug development studies, highly efficacious drugs with reduced side effects are still lacking for human treatment (15). In general, the variability in parameters used in the evaluation of treatment efficacy in both preclinical and clinical studies, such as therapeutic schedule, efficacy against parasite strains, some of which are more resistant to drugs such as VL-10 and Colombiana compared to the Tulahuen strain of *T. cruzi*, and the criteria for cure assessment makes a comparative analysis of the efficacy and potency of these compounds difficult (8, 9). Therefore, one of the main gaps in the evaluation of novel anti-*T. cruzi* compounds is the lack of sensitive assays to detect parasitological cure in preclinical studies and in clinical trials. Hence, there is a great need to discover better cure biomarkers that more closely correlate with the efficacy of novel drug candidates for CD. We have recently reported a non-PCR-based aptamer assay to detect parasite molecules circulating in the blood of infected animals.

We have demonstrated that aptamers (short RNA molecules) can detect parasite antigens circulating in the blood of infected mice using an Enzyme Linked Aptamer (ELA) assay (16, 17). Our previous results showed that ELA assay could detect residual parasitemia in BZ treated mice by providing an overall picture of the infection in the host, suggesting that this assay could be used in drug discovery applications to assess treatment efficacy *in vivo* (18). In the present work, we have further validated the ELA assay using a cure model of *T. cruzi* infected mice treated with Fexinidazole (FZ) (9).

## 2 Materials and methods

### 2.1 Mice and parasites

The *T. cruzi* strains VL-10 (DTU II) and Colombiana (DTU I) were used in this study. VL-10 and Colombiana strains are highly resistant to BZ (9). Female Swiss mice from the Animal Facility at the Federal University of Ouro Preto (UFOP), Minas Gerais State, Brazil, were used in this study. Swiss mice (18–20 g) were inoculated intraperitoneally with 5,000 bloodstream *T. cruzi* trypomastigotes of the VL-10 or the Colombiana strains (9). The mice were divided into groups: infected non-drug treated mice (I group); infected mice treated with BZ (I-BZ group) or infected mice treated with FZ (I-FZ group), and non-infected mice (NI) were used as controls.

### 2.2 Ethics

All procedures and experimental protocols were conducted in accordance with COBEA (Brazilian School of Animal Experimentation) guidelines for the use of animals in research and approved by the Ethics Committee in Animal Research at UFOP (Protocol # 2009/17).

### 2.3 Treatment of *T. cruzi* infected mice

Fexinidazole (1H-imidazole, 1-methyl-2-((4-(methylthio)phenoxy) methyl)-5-nitroimidazole (donated by Drug for Neglected Diseases initiative) was administrated orally in a suspension containing methylcellulose 0.5% w/v, with 5% v/v of polysorbate 80 (Tween 80) (9). Benznidazole (2-nitroimidazole-(Nbenzil-2-nitzo-1-imidazoleacetamide; LAFEPE) was used as the reference treatment in this study and was administered orally in a water suspension with 40.5% methylcellulose (9). The drugs FZ and BZ were administrated with a dose of 300 mg/kg and 100 mg/kg of body weight (mpk), respectively. Drug treatments were started 15 days post-infection (dpi) and administered for 20 days. At 65 dpi, immunosuppression was performed using Cyclophosphamide (N,N-bis(2-chloroethyl)-1,3,2-oxazaphosphinan-2-amine 2-oxide; Genuxal, Asta Medica Oncologica) diluted in ultrapure water and administered intraperitoneally (9). The treatment consisted of three cycles of 50 mg of cyclophosphamide/kg of body weight for four consecutive days with intervals of three days between each cycle. Mice that had detectable parasitemia by microscopy after FZ or BZ treatment were not treated with Cyclophosphamide. The data shown are representative of at least three independent experiments.

### 2.4 Light microscopy

**Figure 1:**
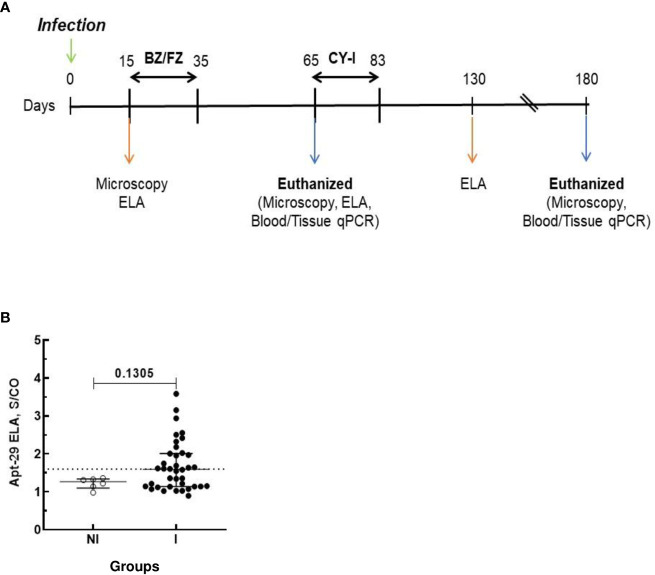
Schematic timeline of *T. cruzi* infection and ELA assay at 15 dpi. Animals were inoculated with 5,000 trypomastigotes of VL-10 *Trypanosoma cruzi* strain. **(A)** Parasitemia was evaluated throughout the experiments by microscopy. Treatment of infected mice with BZ or FZ was started at 15 dpi. Animals that did not present detectable parasitemia by microscopy or blood qPCR after treatment were submitted to immunosuppression consisting of three cycles of 50 mg of cyclophosphamide/kg (CY-I) of body weight for four consecutive days with intervals of three days between each cycle starting at 65 dpi. **(B)** Biomarker levels (S/CO) were obtained by ELA assay from NI (non-infected group, n=6, open circles) and I (infected groups, n=38, solid circles) at 15 dpi before the initiation of treatment. Dotted lines represent the ELA assay cut-off of 1.60. Differences in the median values of the groups were considered significant for p<0.05.

Parasitemia of the animals was evaluated throughout the experiment at the time points shown in the timeline of **Figure 1A** by fresh blood collected from the mouse’s tail and the number of parasites estimated (19).

### 2.5 Real-time PCR

For qPCR, DNA extraction from blood (200 μL) and tissue (~ 20 mg) were performed using a Wizard®Genomic DNA Purification Kit (Promega) as recommended by the manufacturer with modifications (20). DNA was quantified by spectrophotometer (NanoDrop®, Thermo Scientific™), and the concentrations were adjusted to 25 µg/µL.

qPCR reactions were performed in 10 µL containing 50 ng of genomic DNA, 5 µL of SYBR®Green PCR Mastermix (Applied Biosystems), and either 0.35 µM for *T. cruzi* DNA-specific primers or 0.35 µM of murine-specific tumor necrosis factor α (TNF-α) primers. The primers for *T. cruzi* DNA (TCZ-F 5’ -GCTCTTGCCCACAMGGGTGC-3’, where M =A or C and TCZ R 5’ -CCAAGCAGCGGATAGTTCAGG-3’) amplify a 182-bp product (21). Primers for murine TNF-α (TNF-5241 5’-TCCCTCTCATCAGTTCTATGGCCCA-3’ and TNF5411 5’-CAGCAAGCATCTATGCACTTAGACCCC-3’) amplify a 170-bp product (21). The cycling program consisted of an initial denaturation at 95°C for 10 min, followed by 40 cycles of 94°C for 15 s and 62°C for 1 min with fluorescence acquisition at 62°C. Amplification was immediately followed by a melt program with an initial denaturation of 15 s at 95°C, cooling to 60°C for 1 min, and then a stepwise temperature increases of 0.3°C/s from 60 to 95°C. Each 96-well reaction plate contained a standard curve and two negative controls. Negative controls consisted of a reaction with *T. cruzi*-specific or murine-specific primers without DNA and also with blood or tissue DNA from non-infected mice.

Standard curves were generated using DNA extracted from cultured epimastigotes of VL-10 *T. cruzi* strain mixed with DNA extracted from the different tissues collected from uninfected mice (9). Specific molecular standards were generated for each tissue with five serial dilutions at an initial concentration of 1 x 10^6^ parasites/25 μg of tissue DNA. Each DNA sample was quantified in duplicate. The efficiencies of amplification were determined automatically by the StepOneTM Software v2.0 by calculating efficiency (E) (E = 10(−1/slope)) (22). The percentages of positive mice (+ve = positive) by blood or tissue qPCR were calculated by dividing the number of *T. cruzi* PCR positive mice by the total number in the group and multiplying by 100.

### 2.6 Enzyme linked aptamer assay

ELISA plates were coated with 50 μL of mice sera samples diluted 1:200 in phosphate-buffered saline (PBS) and incubated for 1 hour at room temperature. Coated plates were blocked with 1% bovine serum albumin (BSA) in PBS. After discarding the blocking buffer, biotinylated RNA aptamer Apt-29 was added to each well at a concentration of 100 nM prepared PBS (18). After 1 hour of incubation, the plate was washed thrice with PBS to remove unbound aptamers. Streptavidin alkaline phosphatase was added to the wells, and the bound conjugate was detected using 4-Methyllumbelliferyl Phosphate (4-MUP) (Liquid Substrate System, Sigma). Fluorescence was measured at an excitation wavelength of 360 nm and emission wavelength of 440 nm, with a cut-off filter of 435 nm, using a Spectra Max, M5 (Molecular Devices) (18).

ELA data was represented as a Signal to Cut-off (S/CO) ratio calculated by dividing the mean RFU value of each sample by a constant assay cut-off value. The assay cut-off value was established such that the mean S/CO of all mice in the non-infected group was < 1.0. To normalize the RFU data for each ELA plate and to allow comparison between different experiments, *T. cruzi* trypomastigote extract was coated at a final concentration of 200 ng/ml in 1:200 dilution of normal mouse plasma as a positive control for the assay plate. The S/CO of the positive plate control ranged from 3.69 to 3.80 across all experiments. The assay cut-off value was established at 1.6 for *T. cruzi* VL-10 infected mice and at 2.2 for *T. cruzi* Colombiana infected mice.

### 2.7 Statistical analyses

GraphPad PRISM 8.0.1 was utilized for statistical analysis. Unpaired nonparametric Mann-Whitney test was performed to compare the median S/CO values of two groups. Nonparametric Kruskal-Wallis test was performed where the median S/CO of the non-infected control group was compared to that of the infected and the infected drug-treated groups. Kruskal-Wallis test was as gaussian distribution of residuals was not detected. The results are expressed in a scatter plot with bar format (median with interquartile range (25th and 75th)) with individual values represented by symbols. A p-value of < 0.05 was considered statistically significant.

## 3 Results

Swiss mice were infected with either the VL-10 or the Colombiana strain of *T. cruzi* parasites and treated with either FZ or BZ as a comparator. Drug treatment was initiated at 15 days post-infection (dpi) with either 300 mg/kg/day FZ or with 100 mg/kg/day BZ and continued for a total of 20 consecutive days (**Figure 1A**). Light microscopy was performed throughout the experiment for evaluation of the parasitemia in blood. Whole blood (WB) was collected from infected mice pre- and post-drug treatment and pre- and post-immunosuppression with Cyclophosphamide. One fraction of WB was processed for qPCR to detect parasite DNA. Serum generated from another fraction of WB was used in ELA assays to detect biomarker levels at different time points, as indicated in **Figure 1A**. A subset of infected animals was euthanized at 65 dpi, 30 days after the end of the drug treatment. The remaining animals underwent the complete regimen for immunosuppression and were euthanized at 180 dpi.

To determine whether the ELA assay can determine the outcome of drug treatment, plasma from Infected non-drug treated mice (I group); infected mice treated with BZ (I-BZ group), or infected mice treated with FZ (I-FZ group) were evaluated by the ELA assay at 15, 65 and 130 dpi as indicated in **Figure 1A**.

At 15 dpi, mice infected with the VL-10 strain of *T. cruzi* (I group) showed higher biomarker S/CO levels than non-infected (NI) animals. Although the difference between the median S/CO of the two groups was not statistically significant, the data suggests that early during infection (15 dpi), the ELA assay was able to detect infection in 36.8% (14/38) of the infected mice at the assay cut-off of S/CO ≥ 1. 60 (**Figure 1B**). All mice in the infected group presented positive parasitemia by light microscopy at 15 dpi. The fact that the ELA assay did not detect all the infected Swiss mice at 15 dpi may be due to a lesser virulence of the VL-10 strain compared to the Colombiana strain used in our previous studies, which showed detectable biomarker in blood by ELA as early as 7 dpi in mice infected with *T. cruzi* Colombiana parasites (16).

**Figure 2:**
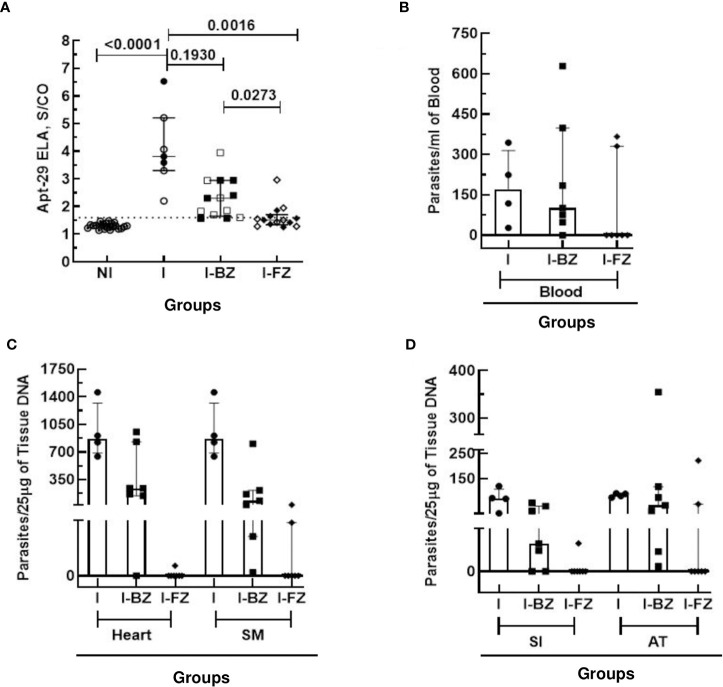
Anti-*T. cruzi* activity of Fexinidazole and Benznidazole in mice infected with VL-10 strain at 65 dpi. **(A)** Biomarker levels (S/CO) were obtained by ELA assay from I (infected untreated control group, n=7), I-BZ (infected treated with BZ, n=14), and I-FZ (infected treated with FZ, n=14) groups at 65 dpi. Dotted lines represent the ELA assay cut-off of 1.60. Differences in the median values of the groups were considered significant for p<0.05. **(B)** Blood qPCR was performed for mice at 65 dpi in the INF (n=7), I-BZ (n=14), and I-FZ (n=14) groups, and tissue qPCR for a subset of mice euthanized at 65 dpi from the INF (n=4), BZ (n=7) and FZ (n=7) groups. **(C, D)** Quantitative analyses of tissue qPCR. SM, Skeletal muscles; SI, small intestine; AT, adipose tissue. The results are expressed in a scatter plot with bar format (median with interquartile range) with individual values represented by symbols. Open symbols in the I-BZ and the I-FZ groups represent mice that were PCR positive.

At 65 dpi, however, the median S/CO of the biomarker levels by ELA assay in the I group (mean S/CO = 4.10 ± 1.3) was statistically significantly higher than the NI and the I-FZ groups. Of significance, biomarker levels in I-BZ were statistically significantly higher than the mice in the FZ treated group (I-FZ), S/CO of 2.30 *vs* 1.61, respectively (**Figure 2A**). These ELA results suggest a treatment efficacy for FZ of 71.42%, with 10 out of 14 mice showing biomarker levels below the ELA assay cut-off compared to 21.43% BZ treatment efficacy (3/14 below the ELA cut-off) (**Figure 2A**; **Table 1**). This finding is consistent with mice infected with VL-10 parasites, a strain significantly resistant to BZ (11, 23). This is also consistent with previous studies showing a cure rate of 88.9% against VL-10 strain in mice treated with FZ during acute phase (9). A randomly chosen subset of the mice were euthanized at 65 dpi (4 out of 7 in the I group, and 7 each out of 14 in the I-BZ and the I-FZ groups, respectively), indicated by open symbols (**Figure 2A**). The superior treatment efficacy of FZ suggested by the ELA results was confirmed by tissue PCR, used as a gold standard assay in this study. In the subset of mice that were euthanized at 65 dpi, tissue and blood PCR results showed that for the FZ treated mice below the ELA assay cut-off, 72% (5/7) were also negative by tissue PCR (solid symbols in the FZ group). The higher biomarker levels in the I-BZ group compared to the I-FZ group and the fact that all BZ-treated mice were tissue PCR positive, show that, as expected for VL-10 parasites, FZ has better treatment efficacy compared to BZ in this animal model. These results show that the ELA assay is able to demonstrate qualitative differences in treatment response to the two drugs and predict treatment failure with BZ.

**Table 1:** Comparative analysis of ELA and qPCR Blood/Tissue at 65 dpi fo VL-10 strain.

Method	Groups		
	I	I BZ	I FZ
ELA	100% +ve (7/7)	79% +ve (11/14)	28% +ve (4/14)
PCR (Blood)	100% +ve (7/7)	79% +ve (11/14)	28% +ve (4/14)
PCR (Heart Tissue)	100% +ve (4/4)	86% +ve (6/7)	14% +ve (1/7)
PCR (Skeletal Muscles)	100% +ve (4/4)	100% +ve (7/7)	28% +ve (2/7)
PCR (Small Intestine)	100% +ve (4/4)	72% +ve (5/7)	14% +ve (1/7)
PCR (Adipose Tissue)	100% +ve (4/4)	100% +ve (7/7)	28% +ve (2/7)

*ELA, Enzyme Linked Aptamer Assay; PCR, Polymerase Chain Reaction; +ve, positive.

I, Infected mice; I BZ, infected treated mice with Benznidazole; I FZ, infected treated mice with Fexinidazole.

At 65 dpi, blood qPCR showed that 100% (7/7) of the mice in the I group, 79% (11/14) in the I-BZ group, and 28% (4/14) in the I-FZ group were positive, indicating residual parasitemia in those mice post-drug treatment in groups I-FZ and I-BZ (**Figure 2B**; **Table 1**). In the mice euthanized at 65 dpi, qPCR was performed to determine tissue resident *T. cruzi* parasites in DNA extracted from heart, skeletal muscle, small intestine, and adipose tissues (**Figures 2C, D**; **Table 1**). The I group, as expected, presented PCR positive (100% (4/4)) from all tissues tested by qPCR. In the I-BZ group, the percent of positivity ranged from 72% (5/7), determined based on DNA extracted from the small intestine, to 100%, determined based on DNA extracted from the adipose tissue and skeletal muscles (**Figures 2C, D**; **Table 1**). In the I-FZ group, 14% (1/7) of the mice were positive for heart and small intestine, and 28% (2/7) for skeletal muscles and adipose tissues tested qPCR (**Figures 2C, D**; **Table 1**).

**Figure 3:**
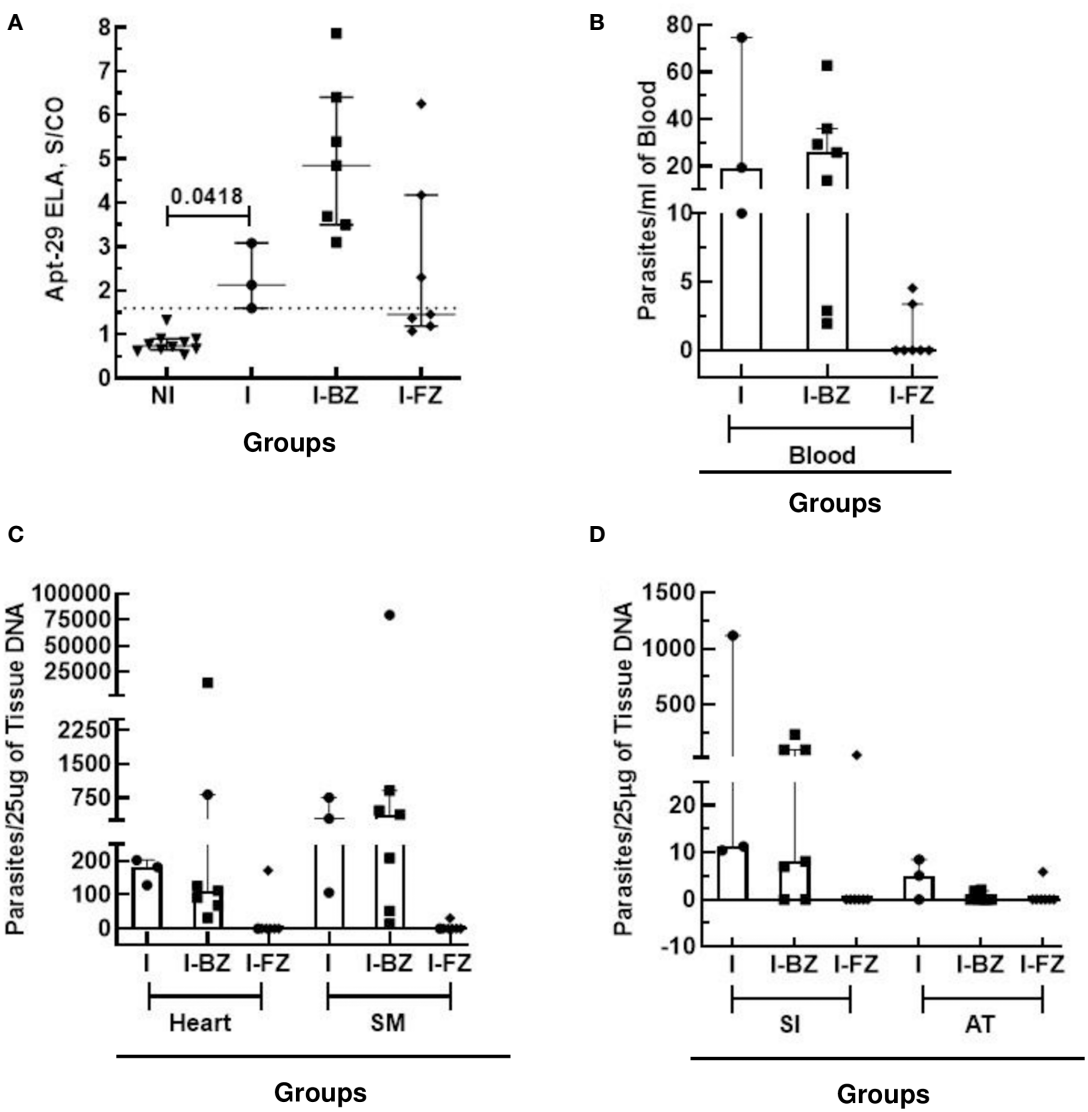
Anti-*T. cruzi* efficacy of Fexinidazole and Benznidazole on mice infected tissue with VL-10 strain at 130 dpi and 180 dpi. **(A)** Biomarker levels (S/CO) were obtained by ELA assay from I (infected untreated control group, n=3), I-BZ (infected treated with BZ, n=7), and I-FZ (infected treated with FZ, n=7) groups at 130 dpi. Dotted lines represent the ELA assay cut-off of 1.60. Differences in the median values were considered significant for p<0.05. **(B)** Blood qPCR was performed for mice at 180 dpi in the INF (n=3), I-BZ (n=7), and I-FZ (n=7) groups, and tissue qPCR for a subset of mice euthanized at 180 dpi from the INF (n=3), BZ (n=7) and FZ (n=7) groups. **(C, D)** Quantitative analyses of tissue qPCR. SM, Skeletal muscles; SI, small intestine; AT, adipose tissue. The results are expressed in a scatter plot with bar format (median with interquartile range) with individual values represented by symbols.

As qPCR following immunosuppression is the most sensitive method available to detect residual *T. cruzi* parasites in drug-treated infected animals (8, 9), we compared the biomarker levels in FZ and BZ treated mice following cyclophosphamide treatment (130 dpi) with blood and tissue qPCR results obtained at 180 dpi (**Figure 3**; **Table 2**). At 130 dpi, the S/CO of the biomarker levels by ELA assay in the I group (not Cyclophosphamide treated) were statistically significantly higher than the NI group (**Figure 3A**), consistent with detectable biomarker levels in chronically infected mice as reported previously (18). The results also showed that 100% (7/7) of mice from I-BZ group and 42% (3/7) of mice from I-FZ were positive by ELA after 130 dpi (**Figure 3A**; **Table 2**). Blood qPCR indicated that 100% of the I (3/3) and I-BZ (7/7) treated mice and 28% (2/7) of the I-FZ groups were qPCR positive, indicating residual parasitemia post-drug treatment (**Figure 3B**; **Table 2**). In the I group, as expected, 100% (3/3) of the mice were positive, with all mice qPCR positive for 3 of the 4 tissues tested. In the I-BZ group, the percent of positivity ranged from 28% (2/7), determined based on DNA extracted from the adipose tissue, to 100% (7/7), determined based on DNA extracted from the heart tissue and skeletal muscles. In the I-FZ group, 14% (1/7) of the mice were positive for all 4 tissues tested qPCR (**Figures 3C, D**; **Table 2**).

**Table 2:** Comparative analysis of ELA (130 dpi) and qPCR Blood/Tissue (180 dpi) for VL-10 strain.

Method	Groups		
	I	BZ	I FZ
ELA	100% +ve (3/3)	100% +ve (7/7)	42% +ve (3/7)
PCR (Blood)	100% +ve (3/3)	100% +ve (7/7)	28% +ve (2/7)
PCR (Heart Tissue)	100% +ve (3/3)	100% +ve (7/7)	14% +ve (1/7)
PCR (Skeletal Muscles)	100% +ve (3/3)	100% +ve (7/7)	14% +ve (1/7)
PCR (Small Intestine)	100% +ve (3/3)	72% +ve (5/7)	14% +ve (1/7)
PCR (Adipose Tissue)	67% +ve (2/3)	28% +ve (2/7)	14% +ve (1/7)

*ELA, Enzyme Linked Aptamer Assay; PCR, Polymerase Chain Reaction; +ve, positive.

I, Infected mice; I BZ, infected treated mice with Benznidazole; I FZ, infected treated mice with Fexinidazole.

Together, the above results show that the ELA assay could demonstrate qualitative differences in treatment response to the two drugs in Swiss mice infected with the VL-10 strain of *T. cruzi*, FZ being more effective than BZ. Further, the ELA assay was able to demonstrate cure by FZ in ~71% of infected mice and could predict treatment failure with BZ in this mouse model.

**Figure 4:**
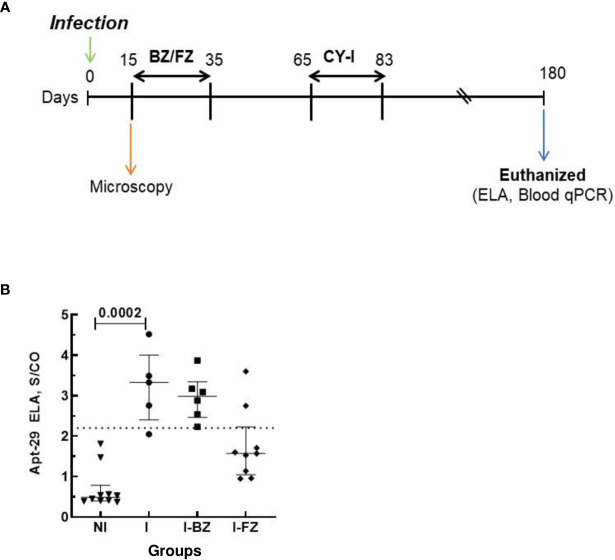
Biomarkers detection in mice infected with Colombiana strain of *T. cruzi* at 180 dpi. Animals were inoculated with 5,000 trypomastigotes of Colombiana strain of *T. cruzi*. **(A)** Parasitemia was evaluated throughout the experiments by microscopy. Treatment of infected mice with BZ or FZ was started at 15 dpi. At 65 dpi, after drug treatment, mice that did not present detectable parasitemia by microscopy or blood qPCR were submitted to immunosuppression as described above. Blood qPCR was performed for mice at 180 dpi in the INF (n=5), I-BZ (n=6), and I-FZ (n=9) groups. **(B)** Biomarker levels (S/CO) were obtained by ELA assay from NI (non-infected group, n=10), I (infected untreated control group, n=5), I-BZ (infected treated with BZ, n=6) and I-FZ (infected treated with FZ, n=9) groups at 180 dpi. Dotted lines represent the ELA assay cut-off of 2.2. Differences in the median values were considered significant for p<0.05.

To determine whether the aptamer-based biomarker detection assay could be used as a universal tool for determining the efficacy of drug treatment in pre-clinical murine models, we tested sera from Swiss mice infected with Colombiana strain of *T. cruzi* and treated with either BZ or FZ, according to the timeline at **Figure 4A**. ELA assay performed at 180 dpi showed that biomarker S/CO from I and I-BZ groups were higher than the I-FZ group, except for 2 mice (**Figure 4B**; **Table 3**). This showed that ELA assay was able to detect the failure of BZ treatment and a cure rate of approximately 80% (2/9 PCR positive) in the FZ treated mice infected with Colombiana strain. Blood qPCR also showed 2 positive mice after FZ treatment (**Table 3**). These results are similar as those obtained with Swiss mice infected with the VL-10 strain of *T. cruzi* described above and suggest the utility of the ELA assay to compare the efficacy of different drugs in various preclinical mice models of CD.

**Table 3:** Comparative analysis of ELA and blood qPCR at 180 dpi for colombiana strain.

Method	Groups		
	I	BZ	FZ
ELA	80% +ve (4/5)	100% +ve (6/6)	22% +ve (2/9)
PCR (Blood)	100% +ve (5/5)	100% +ve (6/6)	22% +ve (2/9)

*ELA, Enzyme Linked Aptamer Assay; PCR, Polymerase Chain Reaction; +ve, positive.

I, Infected mice; I BZ, infected treated mice with Benznidazole; I FZ, infected treated mice with Fexinidazole.

## 4 Discussion

The evaluation of cure for CD is a complicated aspect of its treatment. There is a need for better biomarkers to determine whether a patient treated with an active chemotherapeutic agent is cured. In most cases, the PCR detection of *T. cruzi* DNA in the blood becomes negative post-treatment, while the serological assay remains positive, making it difficult to determine whether the treatment protocol was effective (15). PCR has been proposed as an alternative tool for *T. cruzi* quantification, since it is more sensitive than the traditional parasitological techniques, such as microscopy, xenodiagnoses, and hemoculture (24–27). However, PCR is time-consuming, technically challenging, and presents a high risk of false positive results due to the possibility of carry-over contamination. PCR may also give false negative results, when the parasitemia is very low, such as during the chronic phase of the infection or following drug treatment, due to the limited volume of sample tested.

To determine whether an aptamer-based biomarker detection assay developed previously in our laboratory can be used to evaluate the efficacy of anti-*T. cruzi* drugs in various mice models, mice infected with resistant *T. cruzi* VL-10 and Colombiana strains were treated with either BZ (reference drug) or FZ, and the outcome of these treatments was evaluated by ELA assays, qPCR, and light microscopy. Blood and tissue qPCR were used as gold standard assays to determine the infection status of the mice and to evaluate the ELA assay results. Blood light microscopy was performed to ensure that all mice were infected and to follow the parasite load throughout the infection, although it is well documented that microscopy shows low sensitivity during the chronic phase or after drug treatment. However, we cannot disregard its utility for monitoring the parasitemia during the acute phase of infection and/or when the parasitemia is elevated.

Our data showed that ELA assay was able to detect the failure of BZ treatment in all the mice (100% agreement with PCR data) and 77.7% efficacy of FZ treatment in mice infected with VL-10 strain. The ELA results agreed with blood and tissue qPCR and identified 2 mice not cured after FZ treatment. It was previously demonstrated that BZ induced a strong reduction in the parasite load in blood and tissues, displaying its potent anti-*T. cruzi* activity; however, a relapse of parasitemia was detected in BZ-treated mice showing that this compound was ineffective in inducing parasitological cure in mice infected with VL-10 strain (8). These results agree with other studies showing that the VL-10 strain of *T. cruzi* is resistant to BZ (28). In addition, no cure was observed in dogs or mice infected with VL-10 BZ-resistant strains (20).

Natural resistance of some *T. cruzi* strains to nitro-derivative compounds might explain therapeutic failure in CD. However, drug-induced reduction of the parasite loads in infected tissues can exert positive effects in the clinical evolution of CD by reducing the associated inflammatory processes.

PCR can detect the presence of parasites in blood and tissues of mice with high sensitivity; however, DNA recovery from blood and tissue can be significantly variable due to the volume of sample available for testing, sample storage/treatment, and DNA isolation techniques. At the end of treatment, ELA assay detected BZ treatment failure and identified the two mice that were not cured after FZ treatment, in agreement with data obtained by blood PCR. The ELA assay provides a global picture of parasitemia in the host by detecting molecules secreted/excreted by both trypomastigotes and amastigotes stages of the parasite. In our study, ELA positive drug treated animals continued to harbor *T. cruzi* parasites in the body. Thus, our results provided evidence that ELA assay can be a suitable method to detect a *T. cruzi* infection and for assessing the effectiveness of treatment. Of significance, the ELA assay revealed that FZ was a more effective treatment compared to BZ to treat *T. cruzi* infected mice. This agrees with previous findings, which demonstrated that FZ was superior to BZ or NF in one acute murine infection model with the *T. cruzi* Brazil 32 strain (23). Moreover, high efficacy of FZ was also demonstrated in an animal model where FZ, but not BZ, could reduce myocarditis in FZ-treated infected animals during the chronic phase of *T. cruzi* VL-10 infection (9, 15). FZ is well absorbed when administered orally and broadly distributed to all animal organs and tissues (24). Our study suggests that biomarker detection using ELA could be used to compare and select candidate drugs during the pre-clinical phase of development. This method presents several advantages compared to PCR as the animals do not need to be euthanized, and a biomarker-based approach could yield continuous longitudinal data on the therapeutic effect of the drug. Moreover, ELA assays can be performed using standard ELISA equipment and do not require a highly controlled environment free of potential contaminating DNA, expensive reagents, and complex instrumentation such as PCR or flow cytometry. Further, the ELA assays used in this study detect targets that are conserved in different strains of parasites of *T. cruzi* as shown by our present results using VL-10 and Colombiana strains (18).

## 5 Conclusion

In conclusion, the ELA assay represents a useful new assay in investigations involving experimental chemotherapy and drug screening. Besides, the combination of high sensitivity, low contamination risk, and greater work speed confirm the ELA assay as an innovative tool to support studies involving the course of *T. cruzi* infection in experimental chemotherapy.

## Data availability statement

The raw data supporting the conclusions of this article will be made available by the authors, without undue reservation.

## Ethics statement

The animal study was approved by Ethics Committee in Animal Research at UFOP (Protocol # 2009/17). The study was conducted in accordance with the local legislation and institutional requirements.

## Author contributions

FdA: Conceptualization, Data curation, Formal analysis, Funding acquisition, Investigation, Methodology, Supervision, Validation, Writing – original draft, Writing – review & editing. RN: Conceptualization, Data curation, Formal analysis, Funding acquisition, Investigation, Methodology, Validation, Writing – original draft, Writing – review & editing. AM: Investigation, Methodology, Validation, Writing – review & editing. KG: Investigation, Methodology, Writing – review & editing. LF: Investigation, Methodology, Validation, Writing – review & editing. IC: Investigation, Methodology, Writing – original draft, Writing – review & editing. OAM-F: Conceptualization, Funding acquisition, Resources, Supervision, Writing – review & editing. AD: Conceptualization, Data curation, Funding acquisition, Resources, Writing – original draft, Writing – review & editing. MB: Conceptualization, Funding acquisition, Resources, Supervision, Writing – original draft, Writing – review & editing. AT-C: Conceptualization, Data curation, Funding acquisition, Resources, Supervision, Writing – original draft, Writing – review & editing.
